# Elevated Heterogeneous Nuclear Ribonucleoprotein C Expression Correlates With Poor Prognosis in Patients With Surgically Resected Lung Adenocarcinoma

**DOI:** 10.3389/fonc.2020.598437

**Published:** 2021-01-25

**Authors:** Wei Guo, Qilin Huai, Guochao Zhang, Lei Guo, Peng Song, Xuemin Xue, Fengwei Tan, Qi Xue, Shugeng Gao, Jie He

**Affiliations:** ^1^ Department of Thoracic Surgery, National Cancer Center/National Clinical Research Center for Cancer/Cancer Hospital, Chinese Academy of Medical Sciences and Peking Union Medical College, Beijing, China; ^2^ Department of Graduate School, Zunyi Medical University, Zunyi, China; ^3^ Department of Pathology, National Cancer Center/National Clinical Research Center for Cancer/Cancer Hospital, Chinese Academy of Medical Sciences and Peking Union Medical College, Beijing, China

**Keywords:** heterogeneous nuclear ribonucleoprotein C, lung adenocarcinoma, N6-methyladenosine methylation, prognosis, biomarker, immunohistochemistry

## Abstract

**Background:**

Lung adenocarcinoma (LUAD), as the most common histological subtype of lung cancer, is a high-grade malignancy and a leading cause of cancer-related death globally. Identification of biomarkers with prognostic value is of great significance for the diagnosis and treatment of LUAD. Heterogeneous nuclear ribonucleoprotein C (HNRNPC) is an RNA-binding protein “reader” of N6-methyladenosine (m^6^A) methylation, and is related to the progression of various cancers; however, its role in LUAD is unclear. The aims of this study aims were to study the expression and prognostic value of HNRNPC in LUAD.

**Methods:**

The Oncomine database and gene expression profiling interactive analysis (GEPIA) were used for preliminary exploration of HNRNPC expression and prognostic value in LUAD. LUAD cases from The Cancer Genome Atlas (TCGA) (n = 416) and the Kaplan-Meier plotter database (n = 720) were extracted to study the differential expression and prognostic value of HNRNPC. HNRNPC expression in the National Cancer Center of China (NCC) cohort was analyzed by immunohistochemical staining, and the relationship between HNRNPC expression and survival rate evaluated using the Kaplan-Meier method and log-rank test. Univariate and multivariate Cox regression analyses were used to identify independent prognostic factors. Several pathways that were significantly enriched in the HNRNPC high expression group were identified by Gene Set Enrichment Analysis (GSEA).

**Results:**

Five data sets from the Oncomine and GEPIA databases all supported that HNRNPC expression is significantly higher in LUAD than in normal lung tissue. In TCGA cohort, HNRNPC was highly expressed in LUAD tissues and significantly related to age, sex, smoking history, ethnicity, lymph node metastasis, and TNM staging (*P* < 0.001). High HNRNPC expression was significantly correlated with poor prognosis in the three cohorts (NCC, TCGA, and K-M plotter) (*P* < 0.05). Multivariate Cox regression analysis showed that HNRNPC expression was an independent prognostic factor in both TCGA and NCC cohorts (*P* < 0.05). Further, 10 significantly enriched pathways were identified from TCGA data and 118 lung cancer cell lines in CCLE, respectively.

**Conclusions:**

High HNRNPC expression is significantly related to poor overall survival in patients with LUAD, suggesting that HNRNPC may be a cancer-promoting factor and a potential prognostic biomarker in LUAD.

## Introduction

Lung cancers are highly malignant tumors, with morbidity and mortality ranking first among cancers. Globally, approximately 1.5 million patients die from lung cancer each year, with a mortality rate of more than 25% (1). Lung cancer is divided into small cell lung cancer (SCLC) and non-small cell lung cancer (NSCLC), according to its pathological type, with NSCLC accounting for around 85% of total cases (2). NSCLC includes large cell carcinoma, squamous cell carcinoma, adenocarcinoma, and adenosquamous carcinoma. Currently, lung adenocarcinoma (LUAD) accounts for 35%–40% of total lung cancers, and its incidence is increasing (3). Although great breakthroughs have been made in molecular therapy and immunotherapy, and targeted drugs such as anaplastic lymphoma kinase (ALK), epidermal growth factor receptor-tyrosine kinase inhibitor (EGFR-TKI), B-Raf proto-oncogene serine/threonine kinase (BRAF), ROS proto-oncogene 1 receptor tyrosine kinase (ROS1) and Kirsten rat sarcoma viral oncogene homolog (KARS) have brought survival advantages to LUAD patients with relevant gene mutations, but due to drug resistance and low overall mutation rate, the survival times of LUAD patients remain very short (3–5). Therefore, additional effective biomarkers are needed to enable more patients with LUAD to receive effective treatment and prolong patient survival.

N6-methyladenosine (m^6^A) methylation is a reversible epigenetic modification, present in almost all types of RNA in organisms, mediated by factors including adenosine methyltransferase, demethylase, and RNA-binding protein, and is the most versatile and deeply-studied methylation (6). The rapid development of high-throughput sequencing technology and the gradual expansion of epigenetics research have revealed the role of m^6^A in tumors, where aberrant m^6^A RNA methylation is involved in the occurrence and development of various cancers, including colon cancer (7), acute myeloid leukemia (8), and glioblastoma (9). Heterogeneous nuclear ribonucleoprotein C (HNRNPC) is an RNA-binding protein of the HNRNP family that mainly localizes in the cell nucleus, where it mediates the transfer of multiple RNA transcripts and proteins between the nucleus and the cytoplasm (10). RNA-binding proteins including HNRNPC and YTH domains are considered “readers” of the m^6^A modifications, and can selectively recognize m^6^A mRNA sites to mediate mRNA degradation (11). HNRNPC is crucial for RNA splicing (12, 13), RNA expression (14), sequence-non-specific RNA export (15), regulation of 3’ end processing (16), and translation (17–20). Further, evidence supports roles for HNRNPC in regulation of complex biological processes through a variety of mechanisms and in the promotion of cancer occurrence and development. HNRNPC is abnormally up-regulated in hepatocellular carcinoma, glioblastoma, and melanoma (21–23), and has clear roles in the biological processes underlying ovarian cancer, breast cancer, esophageal squamous cell carcinoma, and bladder cancer (24–27). Nonetheless, the potential of HNRNPC as a tumor biomarker has not been widely studied, particularly in LUAD. To the best of our knowledge, there has been no study of the prognostic value of HNRNPC in patients with LUAD following surgical resection.

Rapid advances in high-throughput sequencing technology have profoundly altered approaches to tumor research. Large quantities of publicly available clinical data are now available, allowing researchers to more effectively study tumor characteristics and have improved our ability to diagnose, treat, and prevent cancer. The Cancer Genome Atlas (TCGA, www.cancer.gov/tcga) is a database established in the United States that collects cancer information, including genomic, epigenomic, transcriptomic, and clinical data. Moreover, there are many other cancer databases available, which can be used to explore novel features of LUAD at the molecular biological level.

In the present study, we applied a variety of bioinformatics tools to analyze the prognostic value of HNRNPC in patients with LUAD, and verified the results in a large independent cohort from our hospital. HNRNPC expression levels were examined in patients with LUAD using public data and independent hospital data, including differences between HNRNPC in tumor and paired normal tissue samples, based on immunohistochemistry (IHC) results. The clinical significance of HNRNPC expression for patient overall survival (OS) was also explored. Kaplan-Meier (KM) curve and Cox multiple regression analyses were used to assess the potential of HNRNPC levels to predict prognosis in LUAD. In addition, we used the Search Tool for the Retrieval of Interacting Genes/Proteins (STRING) and gene set enrichment analysis (GSEA) to identify genes and pathways involving HNRNPC, and infer their biological significance.

## Methods and Materials

### Patient and Tissue Samples

**Figure 1:**
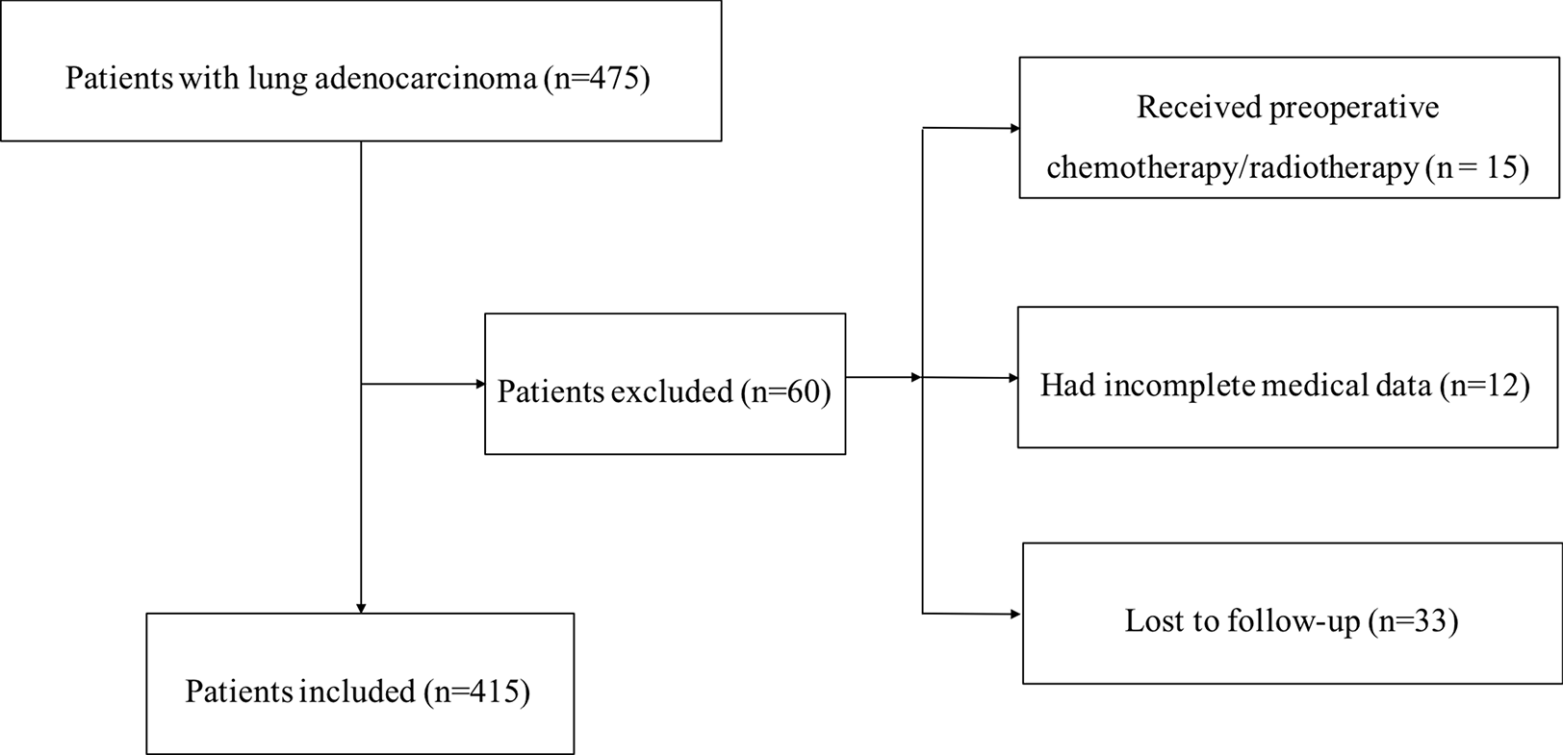
Flowchart of the enrollment process for this study.

The National Cancer Center/Cancer Hospital, Chinese Academy of Medical Sciences (NCC, CAMS, Beijing, China) cohort consisted of 475 patients who had undergone R0 surgical resection and were diagnosed with LUAD between June 2006 and June 2014 in the Department of Thoracic Surgery, NCC. The inclusion criteria were as follows: (1) radical surgery with R0 resection; (2) histologically confirmed LUAD. The exclusion criteria were as follows: (1) if the patients received preoperative chemotherapy and (or) radiotherapy; (2) if the patients lacked detailed clinical information; (3) if the patients lost to regular follow-up. Seventy patients were excluded from this study. The flowchart of the enrollment process is shown in **Figure 1**. Invalid data (n = 60) were excluded, and finally pathological tissue samples and clinical information, including age, sex, smoking history, tumor differentiation status, T stage, lymph node metastasis, and TNM stage were obtained from 415 patients with LUAD. All LUAD tissues were preserved in the NCC biobank and pathologically confirmed by two experienced pathologists.

This research was conducted in accordance with the Declaration of Helsinki and was approved by the Clinical Research Ethics Committee of the National Cancer Center/Cancer Hospital, CAMS.

Patients were followed up every 3–6 months in the outpatient clinic for the first 2 years after surgery, and then annually. Follow-up comprised recording medical history, survival status, physical examination, and chest computed tomography. Final follow-up was March 4, 2019.

### Heterogeneous Nuclear Ribonucleoprotein C Expression and Clinical Information Analyses Using The Cancer Genome Atlas Data

TCGA is a huge cancer genetic data sharing platform containing large amounts of clinical and genomic information to help researchers better understand the genetic basis of cancer and improve their ability to diagnose, treat, and prevent cancer. We downloaded RNA-seq expression profiles and corresponding clinical information for 416 patients with LUAD. RNA expression levels were expressed as reads per kilobase per million mapped reads (RPKM) and converted to transcripts per million (TPM) for comparisons among patients stratified by clinical variables including sex, age, smoking history, T stage, N stage, M stage, TNM stage, ethnicity, and OS data.

### Analysis of Heterogeneous Nuclear Ribonucleoprotein C Expression Using Data From the Oncomine and Gene Expression Profiling Interactive Analysis Databases

The Oncomine database is a website-based cancer-related gene database that contains GEO, TCGA, cancer gene chips, and published research data, and is a central repository storing microarray data, sample data, and analysis results (28). The database contains 715 gene expression datasets, comprising gene expression data from 86,733 cancer and normal tissues. Nine LUAD data sets, including tumors and normal tissues, were extracted for analysis of HNRNPC expression. Gene expression profiling interactive analysis (GEPIA) is a newly-developed interactive web server for analysis of RNA sequencing expression data from 9,736 tumors and 8,587 normal samples from TCGA and GTEx, using a standard processing pipeline (29). GEPIA can be used to analyze tumor/normal differential expression, based on cancer type or pathological stage, as well as for patient survival analysis. In the present study, both Oncomine and GEPIA were used for preliminary investigation of HNRNPC expression. Furthermore, GEPIA was applied for survival analysis of data from TCGA LUAD cohort. Median TPM value was chosen as the cutoff point for grouping.

### Survival Analysis According to Heterogeneous Nuclear Ribonucleoprotein C Expression Using the Kaplan–Meier Plotter Database and National Cancer Center of China Cohort Data

The online survival analysis tool, Kaplan–Meier plotter, integrates multiple cancer data from the Gene Expression Omnibus (GEO), TCGA, and European Genome-phenome Archive (EGA), including lung cancer, breast cancer, gastric and ovarian cancer, and hepatocellular carcinoma data (30). In this research, K-M plotter was used to analyze the correlation between HNRNPC and OS in patients with LUAD, followed by survival curve construction. According to median mRNA expression values, LUAD patients were divided into two groups, and prognostic value analyzed according to hazard ratio (HR) and log-rank p value. Subsequently, data from the NCC cohort were used to verify the prognostic value of HNRNPC.

### Functional Annotation of the Heterogeneous Nuclear Ribonucleoprotein C Protein–Protein Interaction Network and Gene Set Enrichment Analysis

The STRING database (http://string-db.org) aims to provide critical assessment and integration of protein-protein interactions, including direct (physical) as well as indirect (functional) associations (31). A PPI network, centered on HNRNPC, was explored using the “Bioconductor clusterProfiler”, “enrichplot”, and “ggplot2” packages in R (version 3.6.3), and Kyoto Encyclopedia of Genes and Genomes (KEGG) pathways and Gene Ontology (GO) enrichment analysis of molecular functions (MF), biological processes (BP), and cellular components (CC) of the PPI network conducted. An interaction score > 0.4 was considered significant.

The Cancer Cell Line Encyclopedia (CCLE, www.broadinstitute.org/ccle) is a large-scale public database in collaboration between the Broad Institute and the Novartis Institute of Biomedicine and the Genomics Institute of the Novartis Research Foundation. It contains details of 84,434 genes and 1,457 cell lines. The data will help us in-depth research on the molecular characteristics of cancer phenotypes, DNA methylation and potential targets. The project covers most common cancer types, such as lung cancer, liver cancer, stomach cancer, breast cancer and kidney cancer (32). In order to explore the possible molecular mechanisms of HNRNPC in lung cancer cell lines, we downloaded and compiled RNA-seq data of 188 lung cancer cell lines on the CCLE website. Taking the HNRNPC high expression group as a reference, the RNA-seq of these lung cancer cell lines was grouped for subsequent analysis.

GSEA (http://software.broadinstitute.org/gsea/index.jsp) is a powerful algorithm that can identify different gene sets, sort the predefined genes according to the degree of differential expression in two types of sample, and distinguish biological functions or molecular pathways that are significantly aggregated between two different biological states (33). To determine pathways closely related to HNRNPC expression, data from 515 patients with LUAD from TCGA were subjected to GESA. Median HNRNPC expression in the high and low groups were used as standards. Hallmark in GSEA was used to identify predefined gene sets; 5,000 permutations were performed, according to the gene set, to determine p-values. Pathways with p < 0.01 and false discovery rate (FDR) < 0.25 were considered significant, as listed in the Results section. Finally, we performed GSEA on the RNA-seq data of 188 lung cancer cell lines from CCLE to further explore the signal pathways of HNRNPC that are significantly related in cell lines.

### Tissue Microarray Preparation and Immunohistochemistry of Heterogeneous Nuclear Ribonucleoprotein C

Tissue microarrays (TMAs) were prepared from 415 LUAD patient tissue blocks retrieved from the hospital biobank. TMAs were immune-stained using immunohistochemistry methods. Briefly, representative areas of tumor tissue on Hematoxylin and Eosin (H&E) stained sections were identified and labeled by pathologists. TMAs were deparaffinized and rehydrated in sequence, treated with 2 N HCl for 15 min, and then treated with 100 mM Tris HCl (pH 8.5) for 10 min. Samples were then treated with H_2_O_2_ (3%) and goat serum at room temperature for 30 min. After confirmation of blocking, rabbit anti-HNRNPC polyclonal antibody (1:50, HPA051075; Sigma-Aldrich, St. Louis, MO) was added and incubated overnight at 4°C. Then, samples were incubated with polyclonal peroxidase-conjugated anti-rabbit IgG (Zhongshanjinqiao, Beijing, China) for 20 min at room temperature, according to the manufacturer’s instructions.

### Evaluation of Immunostaining

Two experienced pathologists, blinded to the clinical data, independently reviewed TMA IHC staining. The percentage of positive cells and staining intensity of positive cells were used to generate IHC HNRNPC staining scores, calculated based on the percentage of positive cells, as follows: score 0, 0%–25% HNRNPC-positive staining; score 1, 25%–50% HNRNPC-positive staining; and score 2, > 50% HNRNPC-positive staining. Another score was calculated based on the staining intensity of positive cells, as follows: 0, no staining; 1, weak; 2, moderate; and 3, strong. The product of these two scores was the IHC score for each LUAD patient. In the present study, tissues with scores ≤ 1 were classified into the low expression group, and tissues with scores ≥ 2 were classified into the high expression group.

### Statistical Analysis

Nonparametric tests were used to assess correlations between expression of HNRNPC and clinicopathological parameters (a Wilcox test for comparisons between two groups of data and the Kruskal test for three or more groups) in R software (version 3.6.3; The R Foundation for Statistical Computing). HNRNPC expression and clinical data were analyzed by χ^2^ text or Fisher’s exact test. Function enrichment analyses were performed using the Bioconductor package in R. Kaplan-Meier survival analysis and the log-rank test were used to evaluate the prognostic value of HNRNPC. Multivariate Cox regression was applied to identify independent prognostic factors. A p value < 0.05 was considered statistically significant, and the median TPM set as the cutoff value.

## Results

### Aberrant Heterogeneous Nuclear Ribonucleoprotein C Downregulation in Lung Adenocarcinoma

**Figure 2:**
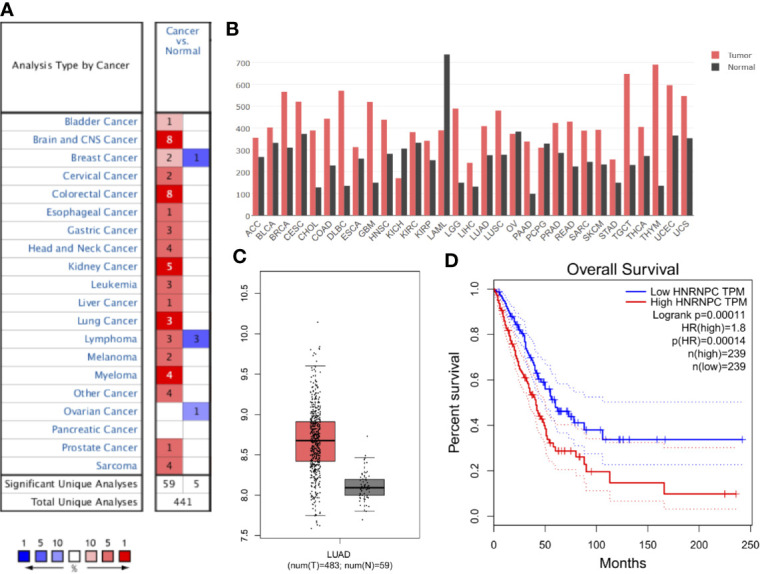
Aberrant up-regulation and prognostic value of HNRNPC in cancer. **(A)** HNRNPC expression in various cancers based on data from Oncomine. Numbers represent the quantity of datasets with significantly (*p* < 0.001) high (red) or low (blue) HNRNPC expression in different cancers. **(B)** HNRNPC expression in various cancer and normal tissues by GEPIA. **(C)** HNRNPC expression in LUAD (red) was higher than that in normal lung tissue (gray) according to GEPIA. **(D)** GEPIA showed that high HNRNPC levels were significantly (*p* < 0.001) associated with poor overall survival in patients with LUAD. GEPIA, gene expression profiling interactive analysis; LUAD, lung adenocarcinoma; HNRNPC, heterogeneous nuclear ribonucleoprotein C; TCGA, The Cancer Genome Atlas.

First, data from the Oncomine database were used to analyze HNRNPC expression in various cancer types. Abnormal HNRNPC expression was retrieved from a total of 441 datasets. Among them, HNRNPC expression levels were significantly increased in tumor tissues in 59 datasets, including brain and central nervous system cancer, breast cancer, cervical cancer, colorectal cancer, esophageal cancer, gastric cancer, head and neck cancer, kidney cancer, lung cancer, myeloma, and many other cancers (**Figure 2A**). In all LUAD datasets, HNRNPC expression was significantly higher in tumor than in normal tissues (**Table 1**). Analysis of HNRNPC expression in TCGA pan-cancer data using GEPIA showed high expression in a variety of tumors (**Figure 2B**). In TCGA LUAD cohort, HNRNPC expression was higher in tumor than normal tissues (**Figure 2C**). Moreover, Kaplan-Meier curves showed that the prognosis of LUAD patients with high HNRNPC expression was significantly worse than that of those with low HNRNPC expression (**Figure 2D**).

**Table 1:** The significant changes of heterogeneous nuclear ribonucleoprotein C (HNRNPC) expression in transcription level between lung adenocarcinoma and normal tissues.

References	P-value	Fold change	Rank (%)	Tumor	Normal
Hou Lung	6.97E-15	1.422	1	45	65
Landi Lung	4.00E-16	1.399	2	58	49
Okayama Lung	6.13E-9	1.412	10	226	20
Stearman Lung	0.002	1.212	15	20	19
Su Lung	0.007	1.247	16	27	30

### Relationship Between Heterogeneous Nuclear Ribonucleoprotein C Expression and Clinicopathological Variables in Patients With Lung adenocarcinoma From The Cancer Genome Atlas

**Figure 3:**
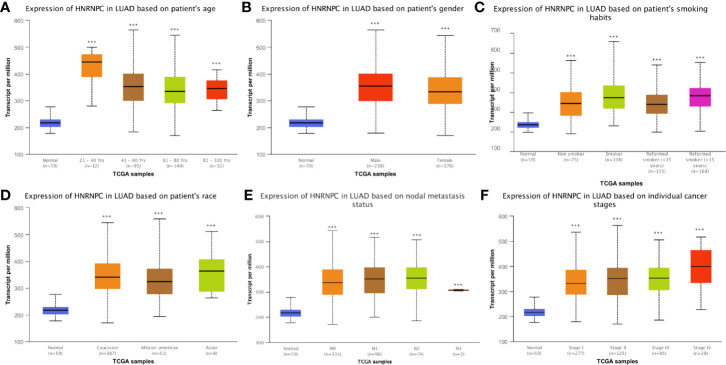
Correlations between heterogeneous nuclear ribonucleoprotein C (HNRNPC) mRNA expression and clinical parameters in The Cancer Genome Atlas (TCGA) cohort: **(A)** age, **(B)** sex, **(C)** smoking history, **(D)** ethnicity, **(E)** N stage, and **(F)** pathological TNM stage; ***p < 0.001.

Next, we determined whether HNRNPC expression in LUAD tissues was associated with clinicopathological features of patients in TCGA dataset. As shown in **Figures 3A–F**, high HNRNPC expression was significantly associated with age (p < 0.001), sex (p < 0.001), smoking history (p < 0.001), ethnicity (p < 0.001), nodal metastasis (p < 0.001), and TNM stage (p < 0.001). In addition, after stratification according to different clinical factors, significant differences were observed among tumor subgroups and the reference/control group (p < 0.05) (**Figures 3A–F**).

To further determine the association between these variables and OS in TCGA datasets, we performed univariate and multivariate Cox logistic regression analyses (**Table 2**). In the univariate model, pT stage (hazard ratio [HR] = 1.168; 95% confidence interval [CI] = 1.076–1.269; p < 0.001), pN stage (HR = 1.374; 95% CI = 1.184–1.594; p < 0.001), TNM stage (HR = 1.207; 95% CI = 1.128–1.29; p < 0.001), and HNRNPC expression (HR = 1.991; 95% CI = 1.478–2.681, p < 0.001) were significantly correlated with OS. Multivariate Cox regression analysis showed that TNM stage (HR = 1.144; 95% CI = 1.042–1.256; p = 0.005), and HNRNPC expression (HR = 1.691; 95% CI = 1.094–2.613; p = 0.018) were independent prognostic factors in the LUAD cohort from TCGA.

**Table 2:** Univariate and multivariate Cox logistic regression analysis of OS in The Cancer Genome Atlas (TCGA) cohorts.

Covariates	Univariate analysis			Multivariate analysis		
	HR	95% CI	*P* value	HR	95% CI	*P* value
Gender (ref. female)	0.947	0.687–1.306	0.741	–	–	–
pT stage (ref. T1–T2)	1.168	1.076–1.269	**<0.001**	1.061	0.968–1.163	0.207
pN stage (ref. N0)	1.374	1.184–1.594	**<0.001**	1.146	0.94–1.397	0.178
pM stage (ref. M0)	0.963	0.878–1.056	0.424	–	–	–
TNM stage (ref. I–II)	1.207	1.128–1.29	**<0.001**	1.144	1.042–1.256	**0.005**
Race (ref. Black)	1.454	0.907–2.329	0.12	–	–	–
Smoked (ref. Never)	0.963	0.687–1.35	0.828	–	–	–
HNRNPC expression (ref. low)	1.991	1.478–2.681	**<0.001**	1.691	1.094–2.613	**0.018**

Bold values mean p value < 0.05.

### Prognostic Analysis of Heterogeneous Nuclear Ribonucleoprotein C Using Kaplan–Meier Plotter and in the National Cancer Center of China Cohort

**Figure 4:**
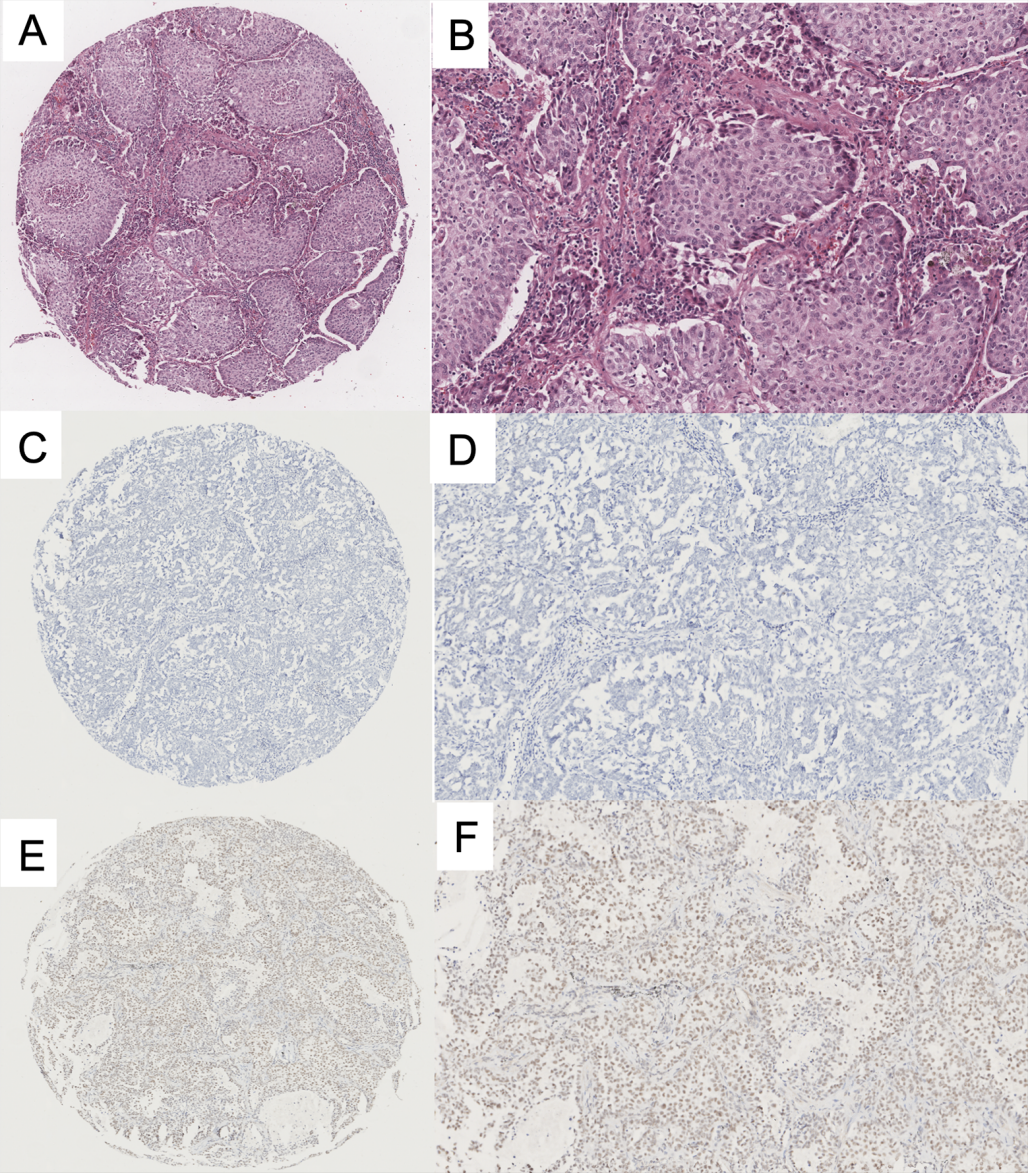
Representative immunohistochemical (IHC) staining of heterogeneous nuclear ribonucleoprotein C (HNRNPC) protein in lung adenocarcinoma (LUAD) TMA. **(A, B)** H&E staining of LUAD tissue. **(C, D)** Negative immunohistochemical staining of HNRNPC in LUAD. **(E, F)** Positive immunohistochemical staining of HNRNPC in LUAD. The three images on the left are low-power fields, those on the right are high-power fields. TMA, tissue microarray; H&E, Hematoxylin and Eosin.

**Figure 5:**
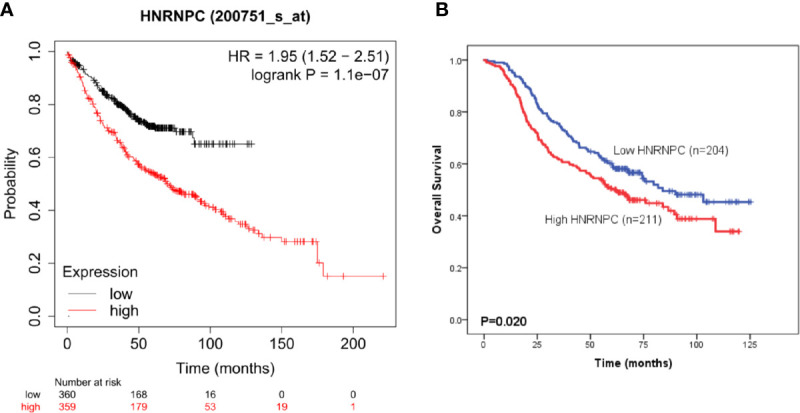
High heterogeneous nuclear ribonucleoprotein C (HNRNPC) expression is significantly associated with poor overall survival (OS) of patients with lung adenocarcinoma (LUAD). **(A)** Kaplan-Meier survival analysis of the relationship between HNRNPC and OS using Kaplan-Meier plotter cohort data. **(B)** Kaplan-Meier survival analysis of the relationship between HNRNPC and OS using the National Cancer Center of China (NCC) cohort data.

To evaluate HNRNPC expression in LUAD specimens, we performed IHC staining on tissue samples from 415 patients from NCC; 211 and 204 patients with LUAD were classified into the high and low expression groups, respectively. Representative photomicrographs of H&E and HNRNPC immunohistochemical staining are shown in **Figure 4**. The Kaplan-Meier plotter database was used to explore the relationship between HNRNPC expression levels and OS of 720 patients with LUAD and HNRNPC probe information. The K-M curve for all patients showed that high HNRNPC levels were significantly correlated with poor OS (p < 1.1e-07) (**Figure 5A**). Subsequently, survival analysis of patients with LUAD from the NCC cohort, by construction of a K-M survival curve, also showed that high HNRNPC expression was significantly correlated with poor OS (p < 0.05) (**Figure 5B**).

### Correlation Between Heterogeneous Nuclear Ribonucleoprotein C Expression and Clinicopathological Parameters in the National Cancer Center of China Cohort

We next determined whether HNRNPC expression in LUAD tissue was associated with patient clinicopathological characteristics. As shown in **Table 3**, other than smoking history (p < 0.05), HNRNPC expression was not significantly associated with age, sex, tumor diameter, tumor differentiation, T stage, N stage, or pathological TNM stage.

**Table 3:** Correlations between heterogeneous nuclear ribonucleoprotein C (HNRNPC) expression and clinicopathological parameters of 415 patients with lung adenocarcinoma (LUAD) in National Cancer Center of China (NCC) cohort.

Category	Cases (number, %)	HNRNPC expression		*P* value
	415 (100%)	Low (204)	High (211)	
Age (years)				0.377
≤60 >60	216 (52.0) 199 (48.0)	111 93	105 106	
Gender				0.080
Male Female	233 (56.1) 182 (43.9)	101 103	132 88	
Smoking				**0.038**
Ever Never	232 (55.9) 183 (44.1)	125 79	107 104	
Tumor length (cm)				0.295
≤4 >4	280 (67.5) 135 (32.5)	143 61	137 74	
Differentiation				0.118
Well Moderate Poor	57 (13.7) 159 (38.3) 199 (48.0)	33 83 88	24 76 111	
T stage				0.444
T1 T2 T3 T4	157 (37.8) 192 (46.3) 41 (9.9) 25 (6.0)	83 94 17 10	74 98 24 15	
N stage				0.545
N0 N1 N2	174 (41.9) 102 (24.6) 139 (33.5)	84 52 68	90 50 71	
TNM stage				0.774
I II III	154 (37.1) 102 (24.6) 159 (38.3)	73 53 78	81 49 81	

Bold values mean p value < 0.05.

Univariate and multivariate Cox regression analyses were also performed using NCC cohort data, to verify the independent prognostic value of HNRPNC expression (**Table 4**). In univariate analysis, OS was significantly associated with age, sex, smoking history, tumor size, tumor differentiation, T stage, lymph node metastasis, pathological TNM stage, and HNRNPC expression (p < 0.05). Further, in multivariate analysis, age, tumor size, lymph node metastasis, pathological TNM stage, and HNRNPC expression remained significantly correlated with OS (p < 0.05).

**Table 4:** Univariate and multivariable analysis of factors associated with overall survival in National Cancer Center of China (NCC) cohort.

	Univariate analysis			Multivariate analysis		
	P value	HR	95%CI	P value	HR	95%CI
Age (≤60, >60years)	**0.002**	1.538	1.173–2.016	**<0.001**	1.669	1.268–2.198
Gender (female, male)	**0.047**	0.756	0.574–0.996	0.785	0.948	0.647–1.390
Smoking (never, ever)	**0.009**	1.430	1.093–1.873	0.330	1.198	0.828–1.732
Tumor length (cm) ≤4 >4	**<0.001**	2.466	1.881–3.233	**<0.001**	1.932	1.413–2.641
Differentiation (well/moderate, poor)	**<0.001**	1.884	1.432–2.478	0.310	1.168	0.865–1.578
T stage (T1/T2, T3/T4)	**<0.001**	1.916	1.389–2.644	0.464	0.863	0.583–1.279
lymph node metastasis (negative, positive)	**<0.001**	2.757	2.026–3.751	**0.002**	1.893	1.268–2.826
TNM stage (I/II, III)	**<0.001**	2.454	1.872–3.217	**0.035**	1.491	1.030–2.159
HNRNPC expression (negative, positive)	**0.020**	1.377	1.051–1.806	**0.011**	1.437	1.089–1.897

### Gene Set Enrichment Analysis and Functional Annotation of an Heterogeneous Nuclear Ribonucleoprotein C Protein–Protein Interaction Network

**Figure 6:**
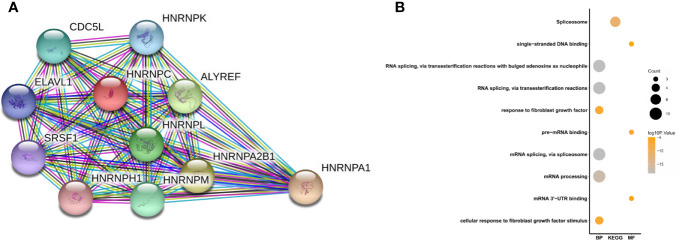
Heterogeneous nuclear ribonucleoprotein C (HNRNPC)-related PPI network construction and biological function analysis. **(A)** PPI network constructed using 10 molecules (CDC5L, HNRNPK, ELAVL1, ALYREF, SRSF1, HNRNPL, HNRNPH1, HNRNPM, HNRNPA2B1, and HNRNPA1) significantly related to HNRNPC. **(B)** GO terms, including biological process (BP) and molecular function (MF), and KEGG pathways analysis. PPI, protein-protein interaction; GO, Gene Ontology; KEGG, Kyoto Encyclopedia of Genes and Genomes.

**Figure 7:**
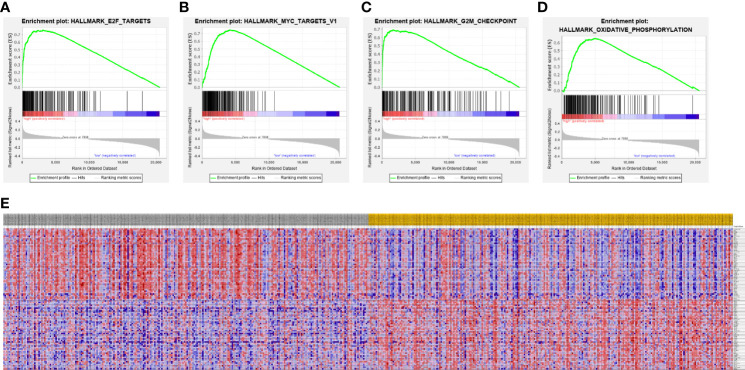
Gene Set Enrichment Analysis according to high expression of heterogeneous nuclear ribonucleoprotein C (HNRNPC). **(A–D)** The top 4 most significantly relevant pathways associated with high expression of HNRNPC. **(A)** E2F targets. **(B)** MYC targets. **(C)** G2M checkpoint. **(D)** Oxidative phosphorylation. **(E)** Heat map of the top 100 genes significantly positively or negatively correlated with HNRNPC levels.

**Figure 8:**
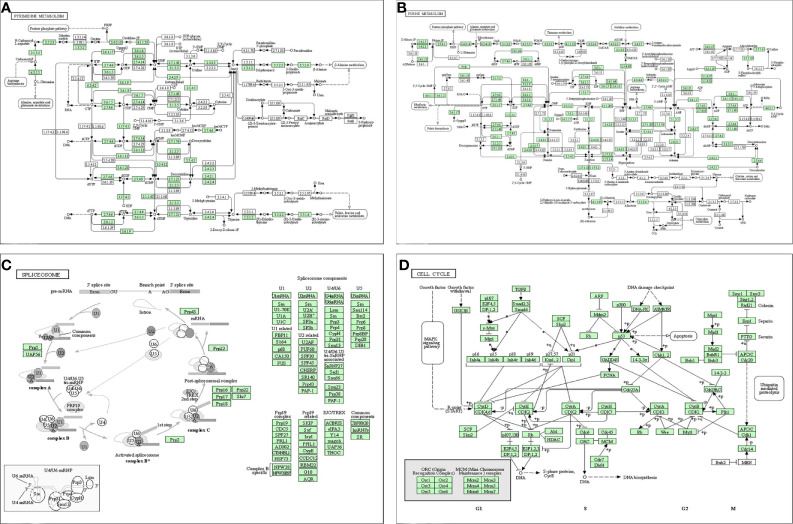
Dissection of heterogeneous nuclear ribonucleoprotein C (HNRNPC)-associated signaling pathways by Gene Set Enrichment Analysis of 188 lung cancer cell lines from the Cancer Cell Line Encyclopedia database (CCLE). **(A)** pyrimidine metabolism. **(B)** purine metabolism. **(C)** spliceosome. **(D)** cell cycle.

A PPI network was constructed from the STRING database to investigate the relationships among 10 molecules that were significantly related to HNRNPC, namely cell division cycle 5 like (CDC5L), heterogeneous nuclear ribonucleoprotein K (HNRNPK), ELAV like RNA binding protein 1 (ELAVL1), Aly/REF export factor (ALYREF), serine/arginine rich splicing factor 1 (SRSF1), heterogeneous nuclear ribonucleoprotein L (HNRNPL), heterogeneous nuclear ribonucleoprotein H1 (HNRNPH1), heterogeneous nuclear ribonucleoprotein M (HNRNPM), heterogeneous nuclear ribonucleoprotein A2/B1 (HNRNPA2B1), and heterogeneous nuclear ribonucleoprotein A1 (HNRNPA1) (**Figure 6A**). To explore the functions of all proteins involved in the PPI network, we performed GO and KEGG functional enrichment analyses; significantly enriched terms are presented in **Figure 6B**. Subsequently, we performed GSEA of HNRNPC using Hallmark gene sets. The results showed that, in samples expressing high levels of HNRNPC, many gene sets were positively enriched, with E2F targets, MYC targets, G2M checkpoint, and oxidative phosphorylation the top four most significantly related biological processes (**Figures 7A–D** and **Table 5**). The top 100 genes that were significantly positive or negatively correlated with HNRNPC expression are presented in **Figure 7E**. In addition, the RNA-seq data of 118 lung cancer cell lines from the CCLE database are divided into two groups according to the average expression level of HNRNPC. As shown in **Figures 8A–D**, GSEA analysis demonstrated that HNRNPC was significantly associated with several KEGG signaling pathways, including pyrimidine metabolism, purine metabolism, spliceosome and cell cycle. The top 10 KEGG pathways enriched by HNRNPC in 118 lung cancer cell lines from CCLE are listed (**Table 6**).

**Table 5:** Top 10 biological processes enriched in lung adenocarcinoma based on heterogeneous nuclear ribonucleoprotein C (HNRNPC).

Rank	Name of pathway	ES	NES	NOM p-value	FDR q-value	FWER p-value
1	E2F_TARGETS	0.76	4.21	0.000	0.000	0.000
2	MYC_TARGETS_V1	0.75	3.98	0.000	0.000	0.000
3	G2M_CHECKPOINT	0.69	3.96	0.000	0.000	0.000
4	OXIDATIVE_PHOSPHORYLATION	0.65	3.40	0.000	0.000	0.000
5	MYC_TARGETS_V2	0.72	3.20	0.000	0.000	0.000
6	MTORC1_SIGNALING	0.57	2.99	0.000	0.000	0.000
7	UNFOLDED_PROTEIN_RESPONSE	0.53	2.63	0.000	0.000	0.000
8	DNA_REPAIR	0.54	2.53	0.000	0.000	0.000
9	REACTIVE_OXYGEN_SPECIES_PATHWAY	0.49	2.08	0.000	0.000	0.000
10	GLYCOLYSIS	0.34	1.82	0.000	0.002	0.001

**Table 6:** Top 10 Kyoto Encyclopedia of Genes and Genomes (KEGG) pathways enriched in 118 lung cancer cell lines from Cancer Cell Line Encyclopedia (CCLE) based on heterogeneous nuclear ribonucleoprotein C (HNRNPC).

Rank	Name of pathway	ES	NES	NOM p-value	FDR q-value	FWER p-value
1	PYRIMIDINE_METABOLISM	0.67	2.52	0.000	0.000	0.000
2	PURINE_METABOLISM	0.82	2.49	0.000	0.000	0.000
3	SPLICEOSOME	0.70	2.39	0.000	0.000	0.000
4	CELL_CYCLE	0.60	2.38	0.000	0.000	0.000
5	RNA_DEGRADATION	0.75	2.37	0.000	0.000	0.000
6	RNA_POLYMERASE	0.81	2.31	0.000	0.000	0.000
7	BASAL_TRANSCRIPTION_FACTORS	0.73	2.25	0.000	0.000	0.000
8	DNA_REPLICATION	0.83	2.17	0.000	0.000	0.001
9	MISMATCH_REPAIR	0.80	2.15	0.000	0.000	0.002
10	NUCLEOTIDE_EXCISION_REPAIR	0.69	2.15	0.000	0.000	0.002

## Discussion

LUAD is a highly heterogeneous malignant tumor with varied pathogenic mechanisms and patient prognoses. Emerging evidence suggests that m^6^A modification is related to tumor proliferation, differentiation, tumorigenesis, invasion, and metastasis, and can contribute to promotion or inhibition of the occurrence and development of malignant tumors (34). To our best knowledge, this is the first study to assess the prognostic value of HNRNPC, a m^6^A methylation related factor, in LUAD.

HNRNPC is an RNA binding protein that has been studied in a variety of tumors; it is important for m^6^A methylation and may be a prognostic marker for certain cancers. Wu and colleagues demonstrated that the tumorigenic effect of HNRNPC on breast cancer MCF7 and T47D cells, where inhibition of HNRNPC expression could effectively reduce cell proliferation and tumor growth *in vivo* (35). Further, down-regulation of HNRNPC can reduce miR-21 expression and decrease AKT serine/threonine kinase (AKT) phosphorylation in ovarian cancer cells, thereby promoting programmed cell death and inhibiting ovarian cancer growth (24). High HNRNPC expression is associated with poor OS and promotes chemoresistance in patients with gastric cancer (36). In addition, HNRNPC overexpression plays a key role in the alternative cleavage and polyadenylation (APA) characteristics of metastatic colon cancer cells. APA occurs in more than half of human genes and is closely related to the formation of mRNA isoforms, thereby changing the stability and coding potential of RNA, leading to a variety of diseases including cancer (37). Further, HNRNPC can interact with p53 directly, degrade p53 in tumor cells, and prevent its activation under normal conditions, which has a negative physiological impact. Knockdown of HNRNPC can improve the stability and activity of p53, with anti-tumor effects (38); however, there have been few studies of HNRNPC in LUAD, and its prognostic value warrants further investigation.

This study involved a large sample, including immunohistochemical staining data, and used bioinformatics technology to explore the clinical significance and prognostic value of HNRNPC expression in LUAD. First, five independent datasets from the Oncomine database consistently demonstrated that HNRNPC levels were significantly higher in LUAD compared with normal tissues, and similar results were found using GEPIA. Subsequently, information for 416 patients with LUAD extracted from TCGA database showed that HNRNPC levels were significantly higher in tumor tissues and an independent prognostic factor for patients with LUAD, indicating that HNRNPC has substantial research value in LUAD. Importantly, K-M plotter analysis of data from our own cohort confirmed that high HNRNPC expression levels were significantly associated with poor OS. Cox regression analysis using NCC cohort data also showed that HNRNPC expression, age, tumor size, lymph node metastasis, and pathological TNM stage were independent prognostic factors for patients with LUAD. In contrast, compared with TCGA cohort, except for smoking history, the expression of HNRNPC in the NCC cohort was not significantly associated with clinicopathological parameters.

There have been few studies of the molecular mechanisms involved in HNRNPC function in LUAD to date. To explore its potential biological functions, we constructed a PPI network with HNRNPC as the core, which showed that mRNA processing and splicing were the main associated biological processes. 10 enriched hallmark HNRNPC pathways were identified by GSEA, including E2F targets, MYC targets v1, G2M checkpoint, and oxidative phosphorylation, among others. E2F activation can promote tumor growth and invasion, and targeting E2F can inhibit lung cancer cell growth (39). Oxidative phosphorylation is key in promoting lung cancer cell metabolism and growth, and many regulatory factors can drive or inhibit lung cancer progression through regulation of oxidative phosphorylation in tumor cells (40, 41). In addition, we performed GSEA on the RNA-seq data of 188 lung cancer cell lines from CCLE to further explore the signal pathways of HNRNPC that are significantly related in cell lines. These specific pathways involving in HNRNPC require further studied *in vivo* and *in vitro*.

The limitations of this study must be acknowledged. Although we used immunohistochemistry data from a large sample cohort from our hospital, the inevitable drawbacks of a single center studies apply to our findings. Samples were drawn from those surgically resected and stored in the biobank; hence, they do not represent the entire LUAD patient population, and underlying molecular biological mechanisms could not be further investigated; additional research into biological function will increase understanding. In conclusion, our data confirm the prognostic value of HNRNPC in LUAD, based on the results of analyses using abundant bioinformatics techniques, and verification of the correlation between HNRNPC and LUAD patient OS by immunohistochemical analysis in a large sample cohort. Our study strongly supports the prognostic value of HNRNPC in LUAD.

## Data Availability Statement

The datasets presented in this study can be found in online repositories. The names of the repository/repositories and accession number(s) can be found in the article/supplementary material.

## Ethics Statement

The studies involving human participants were reviewed and approved by The Clinical Research Ethics Committee of the National Cancer Center/Cancer Hospital, Chinese Academy of Medical Sciences. The patients/participants provided their written informed consent to participate in this study. Written informed consent was obtained from the individual(s) for the publication of any potentially identifiable images or data included in this article.

## Author Contributions

JH, SG, QX, and WG designed the study. WG, QH, GZ, and LG performed the experiments. WG, QH, GZ, PS, XX, and FT analyzed the data. WG, QH, and GZ wrote the manuscript. All authors contributed to the article and approved the submitted version.

## Funding

This work was supported by the National Natural Science Foundation of China (82002451), the Institutional Fundamental Research Funds (2018PT32033), the Ministry of Education Innovation Team Development Project (IRT-17R10), and the Beijing Hope Run Special Fund of Cancer Foundation of China (LC2019B15).

## Conflict of Interest

The authors declare that the research was conducted in the absence of any commercial or financial relationships that could be construed as a potential conflict of interest.
